# Pheromone-sensing neurons regulate peripheral lipid metabolism in *Caenorhabditis elegans*

**DOI:** 10.1371/journal.pgen.1006806

**Published:** 2017-05-18

**Authors:** Rosalind Hussey, Jon Stieglitz, Jaleh Mesgarzadeh, Tiffany T. Locke, Ying K. Zhang, Frank C. Schroeder, Supriya Srinivasan

**Affiliations:** 1 Department of Molecular Medicine and Department of Neuroscience, The Scripps Research Institute, La Jolla, California, United States of America; 2 Dorris Neuroscience Center, The Scripps Research Institute, La Jolla, California, United States of America; 3 Kellogg School of Science and Technology, The Scripps Research Institute, La Jolla, California, United States of America; 4 Department of Biology, University of California, San Diego, La Jolla, California, United States of America; 5 Boyce Thompson Institute and Department of Chemistry and Chemical Biology, Cornell University, Ithaca, New York, United States of America; Stanford University, UNITED STATES

## Abstract

It is now established that the central nervous system plays an important role in regulating whole body metabolism and energy balance. However, the extent to which sensory systems relay environmental information to modulate metabolic events in peripheral tissues has remained poorly understood. In addition, it has been challenging to map the molecular mechanisms underlying discrete sensory modalities with respect to their role in lipid metabolism. In previous work our lab has identified instructive roles for serotonin signaling as a surrogate for food availability, as well as oxygen sensing, in the control of whole body metabolism. In this study, we now identify a role for a pair of pheromone-sensing neurons in regulating fat metabolism in *C*. *elegans*, which has emerged as a tractable and highly informative model to study the neurobiology of metabolism. A genetic screen revealed that GPA-3, a member of the Gα family of G proteins, regulates body fat content in the intestine, the major metabolic organ for *C*. *elegans*. Genetic and reconstitution studies revealed that the potent body fat phenotype of *gpa-3* null mutants is controlled from a pair of neurons called ADL(L/R). We show that cAMP functions as the second messenger in the ADL neurons, and regulates body fat stores via the neurotransmitter acetylcholine, from downstream neurons. We find that the pheromone ascr#3, which is detected by the ADL neurons, regulates body fat stores in a GPA-3-dependent manner. We define here a third sensory modality, pheromone sensing, as a major regulator of body fat metabolism. The pheromone ascr#3 is an indicator of population density, thus we hypothesize that pheromone sensing provides a salient 'denominator' to evaluate the amount of food available within a population and to accordingly adjust metabolic rate and body fat levels.

## Author summary

The central nervous system plays a vital role in regulating whole body metabolism and energy balance. However, the precise cellular, genetic and molecular mechanisms underlying these effects remain a major unsolved mystery. *C*. *elegans* has emerged as a tractable and highly informative model to study the neurobiology of metabolism. Previously, we have identified instructive roles for serotonin signaling as a surrogate for food availability, as well as oxygen sensing, in the control of whole body metabolism. In our current study we have identified a role for a pair of pheromone-sensing neurons in regulating fat metabolism in *C*. *elegans*. cAMP acts as a second messenger in these neurons, and regulates body fat stores via acetylcholine signaling in the nervous system. We find that the population-density-sensing pheromone detected by these neurons regulates body fat stores. Together, we define a third sensory modality, population density sensing, as a major regulator of body fat metabolism.

## Introduction

In relation to fat metabolism and energy balance, the central nervous system plays a more intricate role than historically thought. Initially believed to exert its effects on adiposity predominantly through promoting food intake, several studies have now demonstrated that the underlying neuronal circuits, genetic, molecular and endocrine pathways that regulate body fat reserves in the peripheral metabolic organs are distinct from those that regulate feeding behavior [1–6]. In addition to the pre-eminent role of the mammalian hypothalamus, the sensory nervous system has also been shown to play an important role in regulating whole body metabolism and physiology [7, 8]. Broad sensory dysfunction in humans can be exemplified by ciliopathies such as Bardet-Biedl Syndrome, that leads to profound obesity [9]. In contrast, enhanced sensory environments improve metabolic homeostasis [10]. However, identifying discrete sensory neurons with instructive roles in lipid metabolism has been a challenging undertaking in any system.

In the metazoan *Caenorhabditis elegans*, the nervous system is well-defined at an anatomic and functional level [11, 12]. The sensory nervous system plays a profoundly important role in regulating whole body physiology and lifespan [13–15]. We and others have shown that the sensory nervous system is an important regulator of systemic lipid metabolism [3, 16, 17]. For example, the presence of food, relayed by serotonergic sensory neurons and amplified by the octopaminergic neurons (octopamine is the invertebrate analog of noradrenaline) is one salient input that regulates the magnitude of fat loss in the intestine [3]. The intestine is the predominant metabolic organ for *C*. *elegans*, and expresses all of the genes involved in lipid metabolic processes including fat synthesis, breakdown and its regulation [7, 18, 19]. Furthermore, conserved intestinal fatty acid beta-oxidation has been shown to play a central role in the biosynthesis of the ascarosides, a family of small-molecule pheromones that regulate many aspects of *C*. *elegans* physiology and behavior [20, 21]. Thus, metabolic changes in the intestine effectively encapsulate whole body metabolism. The mechanisms governing lipid metabolism are ancient and well-conserved across metazoans [22–28], therefore *C*. *elegans* offers an excellent platform to identify new genes and molecular mechanisms underlying neuronal control of fat metabolism, using unbiased approaches.

To systematically examine the role of the sensory nervous system in regulating whole body lipid metabolism, we undertook a screen of the 19 (of 21) viable Gα protein mutants for changes in body fat content [29]. This family of heterotrimeric G proteins is well-known to regulate intracellular signaling cascades in response to changes in the environment, which in turn control many aspects of physiology and behavior [30–32]. An added advantage of this family is that the majority of null mutants are viable, and the anatomical locations of these genes have been well-defined. One gene identified from this screen is the Gα protein, GPA-8, the ortholog of the mammalian gustducin proteins that regulates intracellular cGMP. Previous work from our lab has shown that GPA-8 is expressed in the *C*. *elegans* body cavity neurons, and integrates oxygen-sensing with the sensing of internal metabolic state, to drive the rate and extent of fat loss [29, 33]. Thus, we found that environmental oxygen serves as a second physiologically relevant sensory input for the regulation of lipid metabolism.

The most potent 'hit' from our Gα protein screen is called GPA-3, and is a member of the Go/Gi protein family. In this study, we identify the neurons in which GPA-3 functions, define its cellular mechanism of action and the critical downstream neurotransmitter required for its functions in promoting fat loss. In so doing, we define pheromone sensing as a new sensory modality for the regulation of lipid metabolism.

## Results and discussion

### GPA-3 regulates body fat stores via inducing a conserved triglyceride lipase

**Fig 1:**
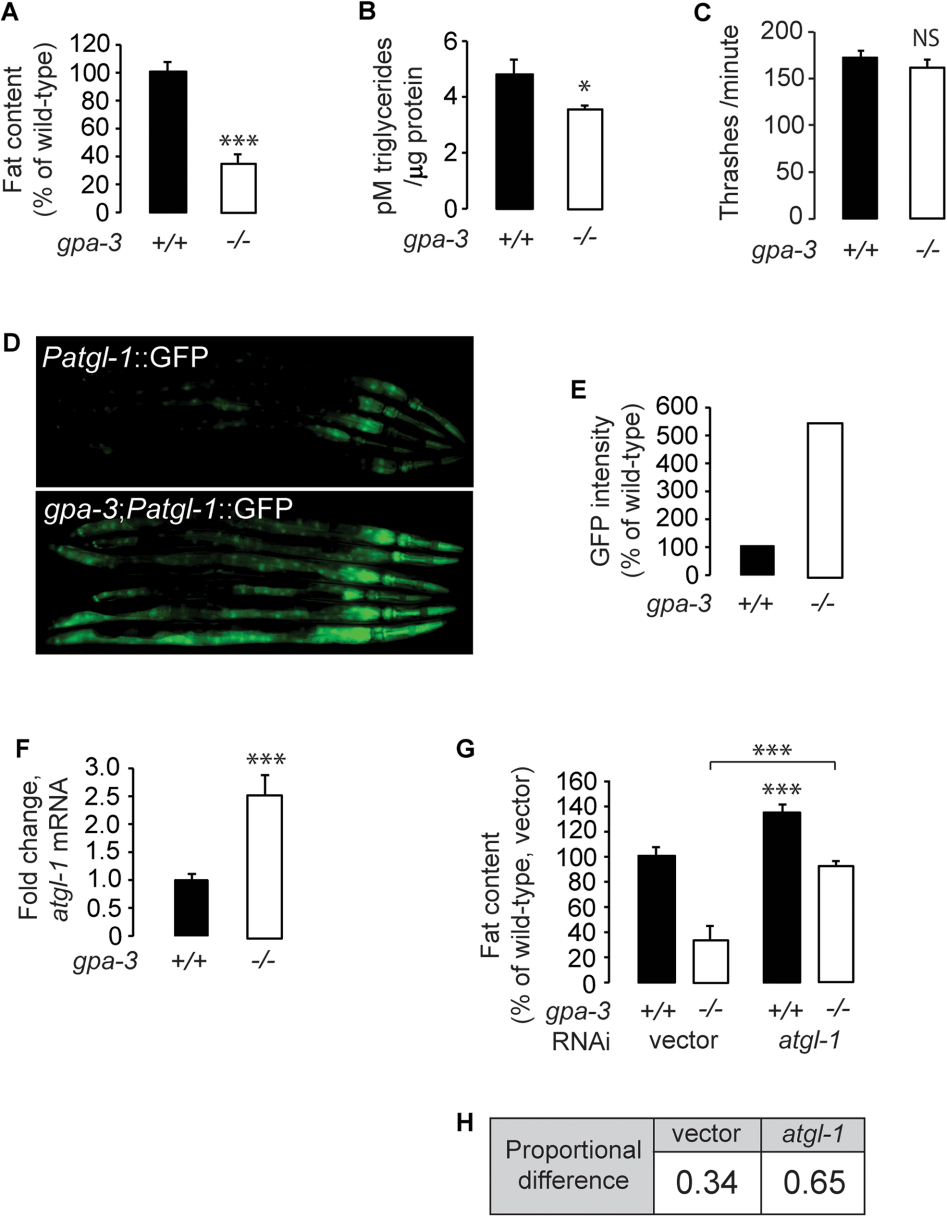
GPA-3 regulates body fat stores via regulating a conserved triglyceride lipase. (A) Wild-type animals and *gpa-3(pk35)* mutants were fixed and stained with Oil Red O. Fat content was quantified for *gpa-3* mutants and is expressed as a percentage of wild-type animals ± SEM (n = 20). ***, p<0.001 by Student’s t-test. See also S1A and S1B Fig. (B) Extracted lipids were quantified by liquid chromatography/mass spectrometry, data were normalized to protein, and quantified using the Pierce BCA Protein Assay kit. *gpa-3* mutants have a significant reduction in triglycerides compared to wild-type animals. *, p<0.05 by Student’s t-test. (C) Thrashing rate was measured for wild-type animals and *gpa-3* mutants. Young adults were individually introduced to M9 buffer and allowed to swim freely for 1 minute to become accustomed to the environment. The number of thrashes was then measured for the next 1 minute. *gpa-3* mutants showed similar motor function to wild-type animals. Data is expressed as number of thrashes per minute ± SEM (n = 15). NS, not significant by Student’s t-test. (D) Representative images are shown of wild-type animals and *gpa-3* mutants bearing an integrated *atgl-1*::*GFP* transgene. (E) The fluorescence intensity of *atgl-1* expression was quantified for 6 randomly selected worms for each genotype and is expressed as a percentage of wild-type animals (F) *atgl-1* mRNA levels were measured by quantitative PCR in *gpa-3* mutants and is expressed as fold change relative to wild-type animals ± SEM (n = 3 biological replicates). ***, p<0.001 by Student’s t-test. (G) Wild-type animals and *gpa-3* mutants were grown on vector or *atgl-1* RNAi containing bacteria and then fixed and stained with Oil Red O. Fat content was quantified for each genotype and condition and is expressed as a percentage of wild-type animals grown on vector RNAi ± SEM (n = 20). Loss of *atgl-1* in *gpa-3* mutants led to a nearly 2-fold increase in fat content relative to wild-type. ***, p<0.001 by Student’s t-test. (H) Data from 1G showing proportional difference in fat content when ATGL-1 is inactivated in wild type animals and *gpa-3* mutants. See also S1C Fig.

*gpa-3(pk35)* null mutants (henceforth *gpa-3*) exhibit a significant decrease in body fat content, as judged by Oil Red O staining of fixed adult animals followed by quantification of lipid droplets in the intestinal cells (Fig 1A and S1A and S1B Fig), and by biochemical extraction of triglycerides from whole adult animals (Fig 1B). The reduced body fat content of *gpa-3* mutants could not be explained by differences in locomotor behavior, which is indistinguishable between wild-type and *gpa-3* mutants (Fig 1C) [34]. Our previous work had identified a highly conserved lipase called adipocyte triglyceride lipase (ATGL-1) that is rate-limiting for fat loss via the conversion of triglycerides to energy by β-oxidation [3]. Work from other groups has shown that the ATGL-1 protein is stabilized by phosphorylation during fasting thus promoting fat loss [35]. *atgl-1* is expressed in the intestine, and is transcriptionally induced in response to neuronal signals that stimulate fat loss. Changes in *atgl-1* transcription are tightly correlated with rates of lipolysis [7, 36], thus changes in *atgl-1* mRNA reflect physiological shifts in energy utilization. Relative to wild-type animals, *gpa-3* mutants have a robust increase in ATGL-1 expression in the intestine (Fig 1D and 1E). Our results indicate that increased fat utilization via induction of triglyceride hydrolysis underlies the reduced body fat of *gpa-3* mutants. To corroborate our experiments using the *atgl-1* reporter line, we conducted qPCR studies and found an approximately 2.5 fold increase in *atgl-1* mRNA in *gpa-3* mutants (Fig 1F). Furthermore, RNA-mediated inactivation of ATGL-1 resulted in a nearly 2-fold suppression of fat loss in the *gpa-3* mutants (Fig 1G and 1H and S1C Fig). Together, these results show that increased triglyceride hydrolysis is one major mechanism underlying the decreased body fat stores of *gpa-3* mutants.

### GPA-3 regulates body fat via cAMP-mediated signaling from amphid sensory neurons

**Fig 2:**
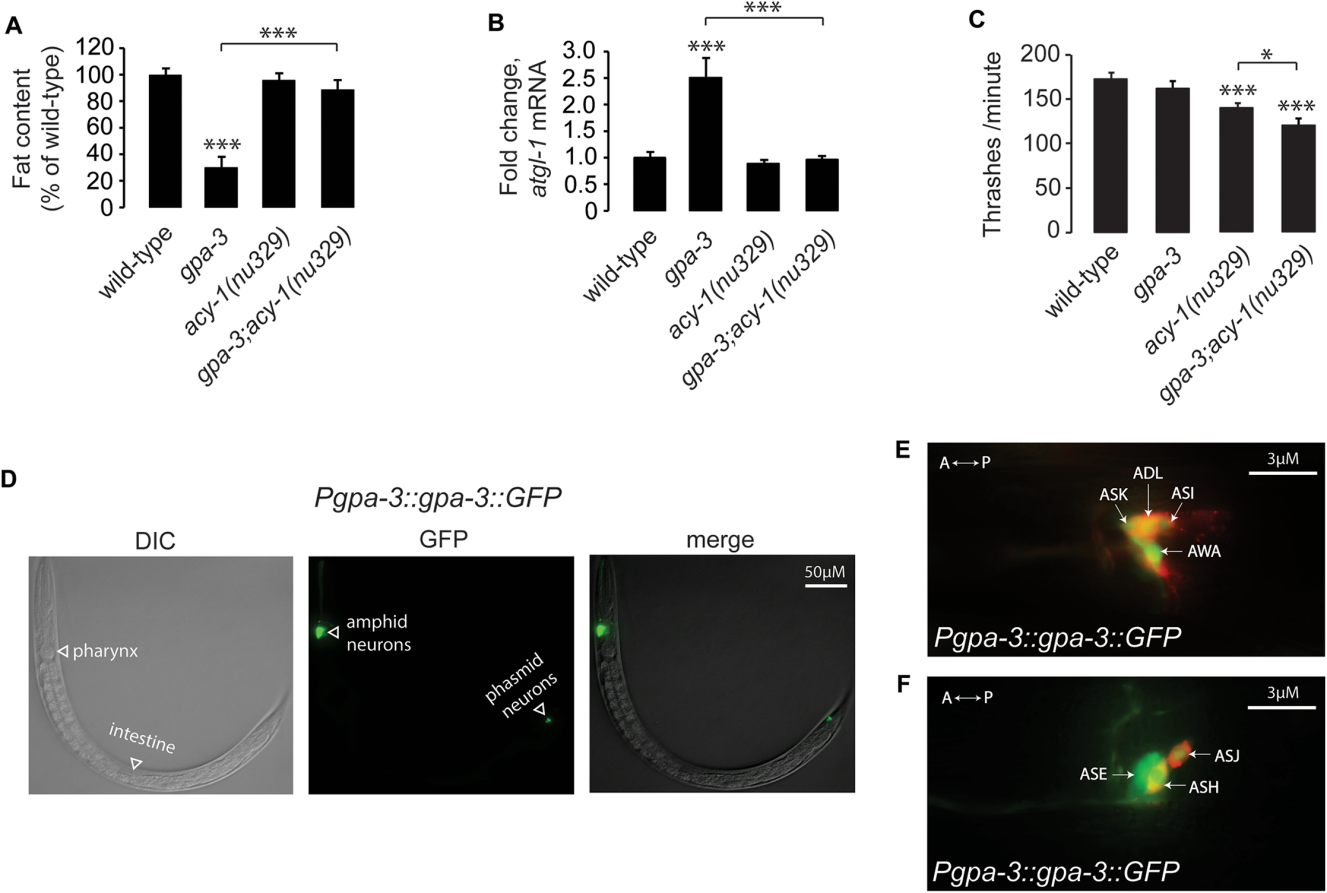
GPA-3 regulates body fat via cAMP-mediated signaling from amphid sensory neurons. (A) Animals were fixed and stained with Oil Red O. Fat content was quantified for each genotype and is expressed as a percentage of wild-type animals ± SEM (n = 20). ***, p<0.001 by one-way ANOVA. See also S2C Fig. (B) *atgl-1* mRNA levels were measured by quantitative PCR in *gpa-3*, *acy-1(nu329)* and *gpa-3;acy-1* mutants and is expressed as fold change relative to wild-type animals ± SEM (n = 3 biological replicates). ***, p<0.001 by one-way ANOVA. (C) Thrashing rate was measured for each genotype. Although *gpa-3* mutants show similar motor function to wild-type animals, *acy-1(nu329)* and *gpa-3;acy-1* mutants. Data is expressed as number of thrashes per minute ± SEM (n = 15). *, p<0.05; ***, p<0.001 by one-way ANOVA. (D) Representative images showing the expression of GPF in transgenic animals bearing a transgene driving *gpa-3* expression with the endogenous promoter. DIC (left panel), GFP (central panel) and a merged representation of these 2 images (right panel). GPA-3 is solely expressed in the nervous system and not in the intestine, where its metabolic phenotype manifests. (E, F). Using DiI staining (red), a method used to identify *C*. *elegans* sensory neurons, we found that GPA-3 is expressed in 9 bilaterally symmetric pairs of amphid sensory neurons with ciliated endings that are directly exposed to the environment. A, anterior; P, posterior.

GPA-3 is orthologous to the mammalian cAMP-regulating Gαo/i class [31], sharing 73% similarity (7e^-139^). Gαo/i family members are known to regulate intracellular cAMP via inhibition of adenylyl cyclases. The *C*. *elegans* cAMP adenylyl cyclase ACY-1 is expressed in neurons, and viable loss-of-function *(nu239)* mutants are available [37]. Although the *acy-1(nu329)* mutants did not show an appreciable difference in body fat (Fig 2A and S2C Fig), removal of *acy-1* in the *gpa-3* mutants resulted in a near-complete suppression of the *gpa-3* body fat phenotype. To provide a second, molecular readout of the fat loss, we measured *atgl-1* mRNA by qPCR, and found that the *gpa-3*-mediated induction of *atgl-1* in the *gpa-3* mutants was also suppressed in the *gpa-3;acy-1* double mutants (Fig 2B). Thus, ACY-1 function is required downstream of GPA-3 in the regulation of body fat via the induction of ATGL-1-mediated lipolysis. *acy-1* mutants have a mild locomotor defect and *gpa-3;acy-1* double mutants have a statistically significant additive effect (Fig 2C). However because *gpa-3* mutants themselves do not have a locomotor phenotype, these effects are non-specific to the *gpa-3* fat regulatory pathway. To determine the direction of the effect of increased cAMP on body fat, we exogenously administered a non-hydrolyzable analog of cAMP called 8-Bromo-cAMP (8-Br-cAMP), which led to a dose-dependent decrease in body fat stores (S2A Fig). Together, our results indicate that GPA-3 controls fat utilization through inhibition of the adenylyl cyclase ACY-1, and the resultant regulation of cAMP concentrations (S2B Fig).

Examination of our *gpa-3*-expressing transgenic lines showed that GPA-3 is solely expressed in the nervous system and not in the intestine, where its metabolic phenotype manifests (Fig 2D). GPA-3 is expressed in 9 bilaterally symmetric pairs of amphid sensory neurons with ciliated endings that are directly exposed to the environment: ADF, ADL, ASE, ASG, ASH, ASI, ASJ, ASK, and sporadically in AWA (Fig 2E and 2F), confirming previous observations [38, 39]. The localization of GPA-3 to the amphid sensory neurons suggests that the subset of neurons from which GPA-3 regulates body fat either directly or indirectly regulate a long-range neuroendocrine factor that acts in the intestine to elicit fat loss.

### GPA-3 functions in ADL amphid sensory neurons to regulate intestinal fat utilization

**Fig 3:**
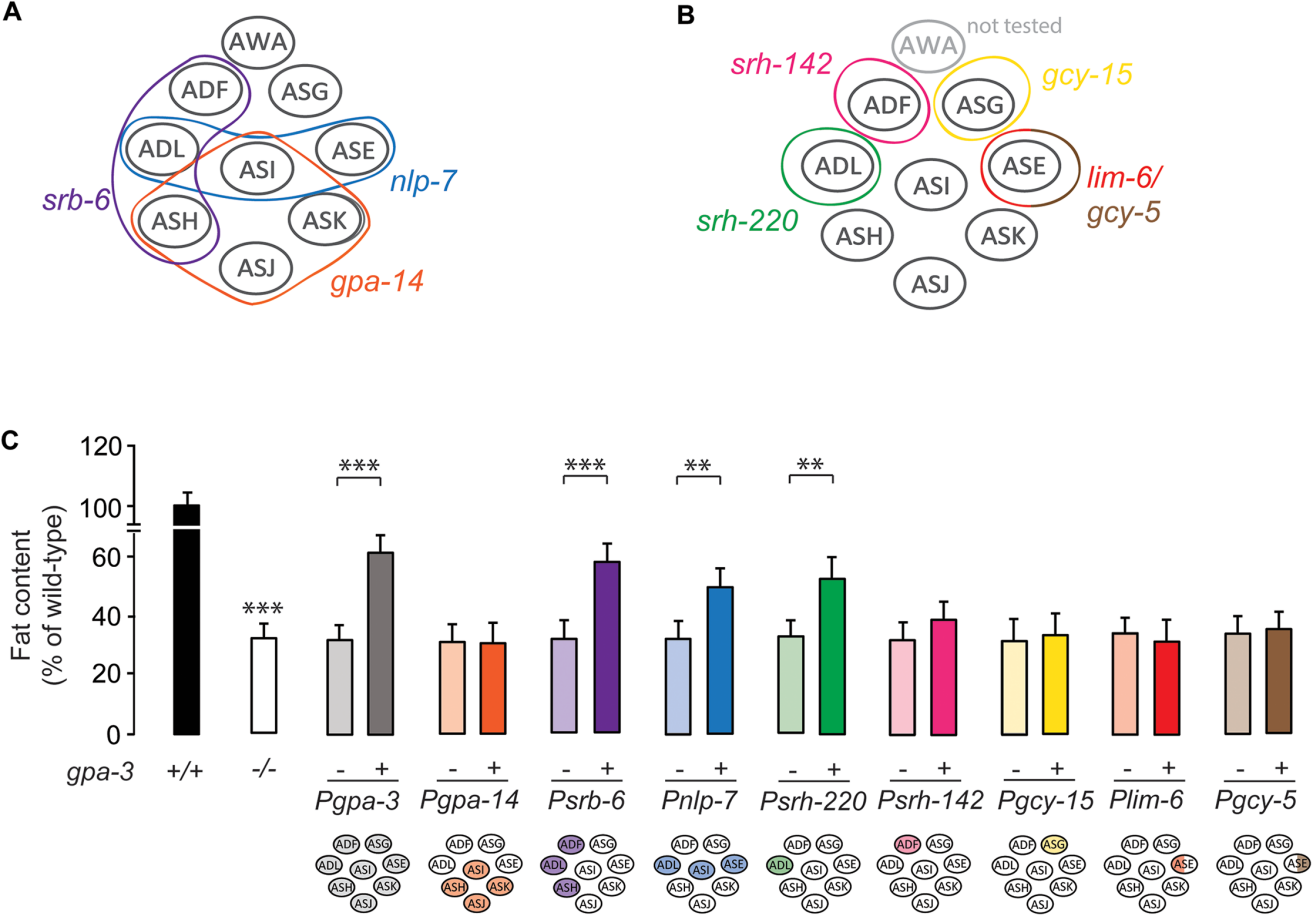
GPA-3 functions in ADL amphid sensory neurons to regulate intestinal fat utilization. (A-B) Model depicting the transgenic rescue strategy utilized to restore *gpa-3* cDNA, first in subsets of amphid neurons (A), and then in individual neuron types (B). (C) *gpa-3* mutants bearing *gpa-3* expression using the indicated promoter were fixed and stained with Oil Red O. Relative to non-transgenic controls (-, light bars), transgenic animals (+, dark bars) bearing the *gpa-3* transgene in ADL neurons restored body fat content. Data are expressed as a percentage of body fat in wild-type animals ± SEM (n = 12–16). **, p<0.01; ***, p<0.001 by one-way ANOVA. See also S3A Fig.

To identify the neurons in which GPA-3 acts to regulate fat stores in the intestine, we generated expression constructs to drive *gpa-3* cDNA in subsets of amphid neurons in which *gpa-3* is normally expressed. The transgenic rescue strategy is given in Fig 3A and 3B. In *gpa-3* null mutants, restoration of *gpa-3* cDNA using either 5kb or 7kb of endogenous *gpa-3* upstream regulatory regions, gave significant restoration of intestinal fat content (Fig 3C and S3A Fig), confirming that GPA-3 functions in sensory neurons to regulate intestinal fat stores. However, we noted that in the *gpa-3* transgenic animals, neither the 5kb nor the 7kb promoter were sufficient to confer a complete restoration of body fat stores (S3A Fig), which prompted us to examine the feeding behavior of *gpa-3* mutants. Relative to wild-type animals, *gpa-3* mutants displayed ~15–20% decrease in food intake (S3B Fig). However, we found that re-expression of *gpa-3* under either 5kb or 7kb promoters did not restore food intake to wild-type levels (S3B Fig).

We next wanted to test whether *acy-1* mutants suppressed the decreased food intake of *gpa-3* mutants, and accordingly measured food intake in the relevant mutants. We found that *acy-1* mutants also displayed decreased food intake to a similar extent as the *gpa-3* mutants. However, the *gpa-3;acy-1* double mutants resembled either single mutant alone (S3C Fig). Thus, unlike the suppression of GPA-3-mediated fat loss (Fig 2A) or its induction of *atgl-1* (Fig 2B), *acy-1* mutants do not suppress GPA-3-mediated food intake.

We wanted to further examine the role of GPA-3 in specific subsets of neurons. Accordingly, we devised a transgenic rescue strategy that allowed us to include or exclude a role for GPA-3 in subsets of neurons in which it is expressed (Fig 3A). In *gpa-3* null mutants, restoration of *gpa-3* cDNA expression using the *srb-6* (ADL, ADF, ASH) and *nlp-7* (ADL, ASI, ASE) promoters, but not the *gpa-14* (ASH, ASI, ASK, ASJ) promoter significantly restored intestinal fat content (Fig 3C). This combinatorial strategy first eliminated a role for GPA-3 in ASI, ASH, ASK and ASJ neurons and second, identified a potential role for the ADL neurons because it is the only neuron pair that overlaps between the two rescuing promoters, *srb-6* and *nlp-7*. We next drove *gpa-3* cDNA expression in the individual neurons ADL, ASG and ASE using neuron-specific promoters (Fig 3B; the AWA neurons were not tested). Restoration of *gpa-3* in the ADL neurons alone significantly restored body fat stores in the *gpa-3* null mutants (Fig 3C). Thus, GPA-3 function in the ADL neurons regulates body fat stores. Although the transgenic rescue strategy revealed a clear role for GPA-3 in controlling body fat in subsets of neurons, in no case were we able to restore the feeding phenotype of *gpa-3* mutants, including the endogenous promoter that was sufficient to restore fat stores (Fig 3C and S3A and S3B and S3D Fig). These results suggest the possibility that background effects unrelated to the *gpa-3* gene contribute to the reduced feeding phenotype in these mutants, despite the *gpa-3* mutant having been outcrossed 7 times. Another possibility is that although unusual in *C*. *elegans* [40], additional regulatory elements further upstream from the chosen 7kb *gpa-3* promoter region may play a role in controlling *gpa-3* expression. However, our data also suggest that the reduced food intake only accounts for a small percentage of the net change in body fat stores, because expression of *gpa-3* in the ADL neurons significantly restores body fat stores without altering food intake (Fig 3C). Together our data suggest that *gpa-3* functions in the ADL neurons to regulate fat content, independent of changes in food intake or locomotion.

### Enhanced cAMP production in ADL neurons decreases intestinal fat

**Fig 4:**
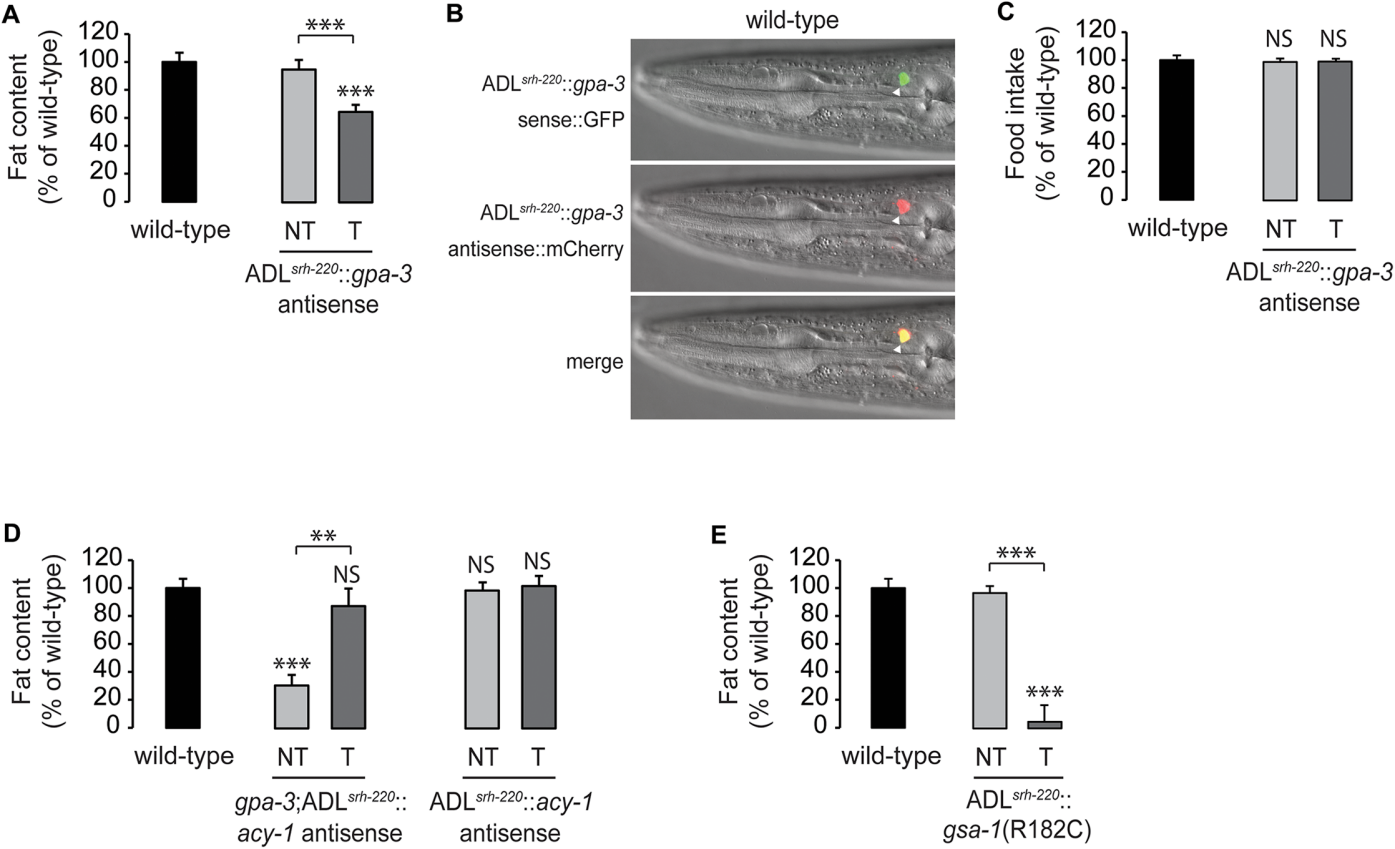
Enhanced cAMP production in ADL neurons decreases intestinal fat. (A) Wild-type animals bearing antisense-mediated inactivation of *gpa-3* expression in ADL neurons using the *srh-220* promoter were fixed and stained with Oil Red O. Relative to non-transgenic controls (NT, light gray bar), transgenic animals (T, dark gray bar) bearing *gpa-3* antisense in ADL neurons had a significant decrease in body fat. Data are expressed as a percentage of body fat in wild-type animals ± SEM (n = 20). ***, p<0.001 by one-way ANOVA. See also S4A Fig. (B) Representative images showing the expression of *gpa-3* sense::GFP (upper panel), *gpa-3* antisense::mCherry (central panel) and their co-localization in ADL (merged, lower panel). (C) Food intake in wild-type animals bearing antisense-mediated inactivation of *gpa-3* expression in ADL neurons. Data are expressed as a percentage of wild-type animals ± SEM (n = 10). NS, not significant by one-way ANOVA. (D) Wild-type animals and *gpa-3* mutants bearing antisense-mediated inactivation of *acy-1* expression in ADL neurons using the *srh-220* promoter were fixed and stained with Oil Red O. Relative to non-transgenic controls in the *gpa-3* background (NT, light gray bar), transgenic animals (T, dark gray bar) bearing *acy-1* antisense in ADL neurons restored body fat content to wild-type. In wild-type animals, there was no difference in fat content of non-transgenic (NT, light gray bar) and (T, dark gray bar) transgenic animals. Data are expressed as a percentage of body fat in wild-type animals ± SEM (n = 20). NS, not significant; **, p<0.01; ***, p<0.001 by one-way ANOVA. See also S4B Fig. (E) Wild-type animals bearing *gsa-1*(R182C), a dominant, gain-of-function mutation of *C*. *elegans* Gαs, in ADL neurons were fixed and stained with Oil Red O. Relative to non-transgenic controls (NT, light gray bar), transgenic animals (T, dark gray bar) bearing *gsa-1*(R182C) in ADL neurons had a significant decrease in body fat. Data are expressed as a percentage of body fat in wild-type animals ± SEM (n = 14–20). ***, p<0.001 by one-way ANOVA. See also S4C Fig.

To determine the necessity of GPA-3 in the ADL neurons for the regulation of body fat, we conducted antisense mediated inhibition experiments [41] using an ADL-specific promoter. Inactivation of *gpa-3* in the ADL neurons lowered fat content to 65% of that seen in wild-type animals (Fig 4A and 4B and S4C Fig). Notably, eliminating *gpa-3* in the ADL neurons in an otherwise wild-type background did not alter food intake (Fig 4C), reinforcing our observations that *gpa-3*-mediated regulation of body fat via the ADL neurons occurs independently of feeding. Together with the transgenic rescue experiments, we find that GPA-3 expression in ADL neurons is necessary and sufficient to maintain body fat stores.

Our genetic epistasis experiments (Fig 2A and 2B and S2A Fig) suggested that *gpa-3* negatively regulates *acy-1* to control intracellular cAMP. We next wanted to determine the extent to which this signaling pathway functions in the ADL neurons. Accordingly, we inactivated *acy-1* solely in the ADL neurons in the *gpa-3* mutant background using antisense inhibition. Relative to non-transgenic controls, inactivation of *acy-1* selectively in the ADL neurons led to a significant suppression of the decreased body fat of the *gpa-3* mutants, resulting in body fat content similar to wild-type levels (Fig 4D and S4B Fig). As seen with global *acy-1* loss (Fig 2A), inactivation of *acy-1* specifically in the ADL neurons also did not appreciably alter fat stores (Fig 4D and S4B Fig). Together, these experiments reveal a role for GPA-3 as a negative regulator of ACY-1 and intracellular cAMP in the ADL neurons, for the control of body fat stores. In *C*. *elegans*, the stimulatory Gαs that activates adenylyl cyclase to increase intracellular cAMP [42] is called GSA-1. To test the prediction that enhanced cAMP production in ADL neurons decreases body fat, we selectively expressed *gsa-1*(R182C), a dominant, gain-of-function mutation of *C*. *elegans* Gαs [43] in the ADL neurons, which resulted in a near-complete loss of intestinal fat (Fig 4E and S4C Fig). Thus, enhanced cAMP signaling in the ADL neurons stimulates fat loss in the intestine, and the cAMP second messenger in ADL neurons is instructive for the control of fat stores in the intestine.

### Cholinergic signaling drives GPA-3-mediated fat loss

**Fig 5:**
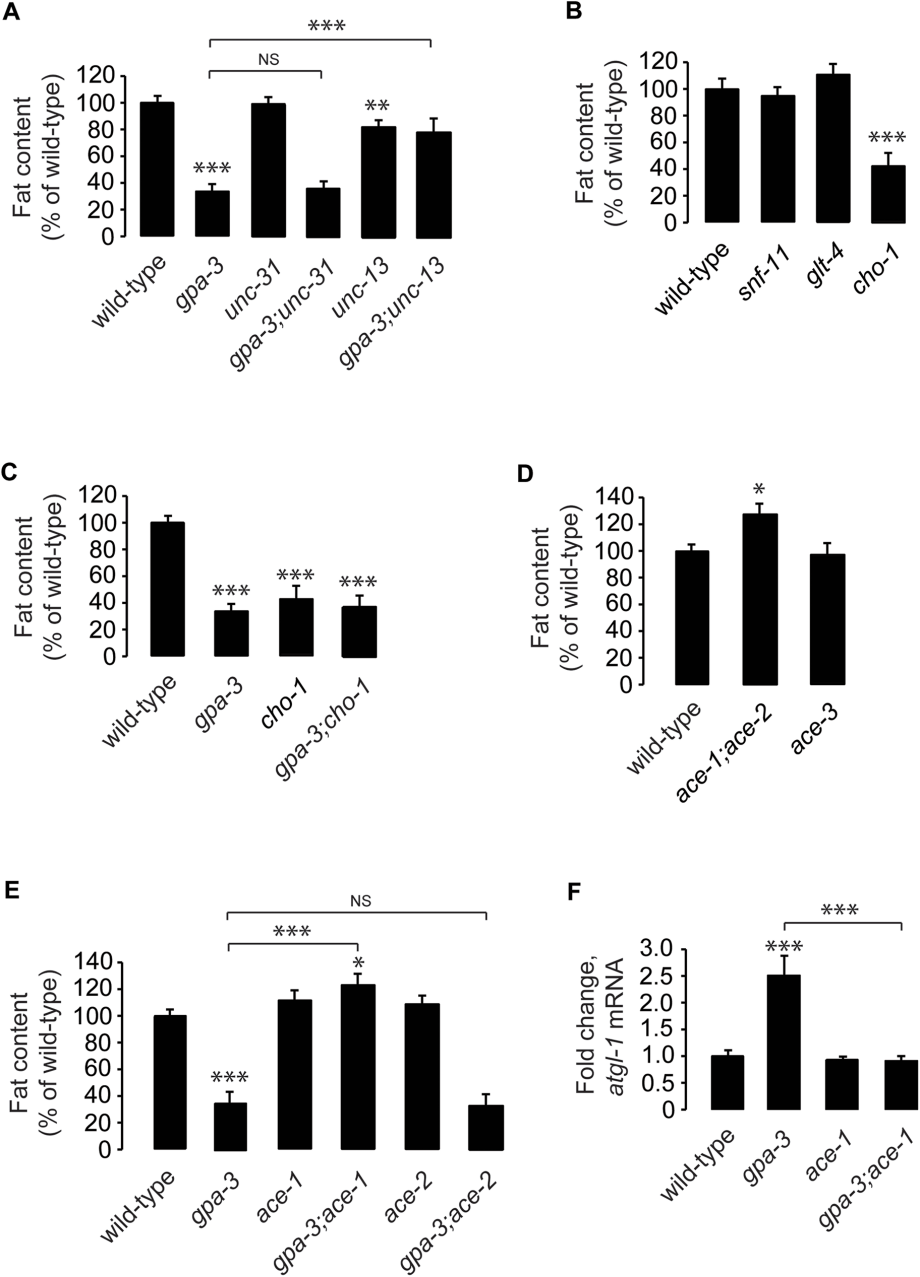
Cholinergic signaling drives GPA-3-mediated fat loss. (A-E) Animals were fixed and stained with Oil Red O. Fat content was quantified for each genotype as indicated, and is expressed as a percentage of wild-type animals ± SEM (n = 14–20). NS, not significant; *, p<0.05; **, p<0.01; ***, p<0.001 by one-way ANOVA. See also S5A–S5E Fig. (F) *atgl-1* mRNA levels were measured by quantitative PCR in *gpa-3*, *ace-1* and *gpa-3;ace-1* mutants and is expressed as fold change relative to wild-type animals ± SEM (n = 3 biological replicates). ***, p<0.001 by one-way ANOVA.

Information from the ADL neurons to the intestine could be relayed either directly via the release of a neuroendocrine factor, or indirectly via modifying the properties of other neurons. These possibilities can be distinguished in the following way: long-range neuropeptides and neuromodulators are localized to dense core vesicles, which require the conserved Calcium-dependent Activator Protein for Secretion (CAPS, UNC-31 in *C*. *elegans*) for fusion with the plasma membrane [44–46]. On the other hand, the canonical neurotransmitters (acetylcholine, ACh; γ-amino butyric acid, GABA; and glutamate) are localized to small clear synaptic vesicles, which require a protein called UNC-13 (MUNC-13 in mammals) for fusion at the synapse [47, 48]. Thus, loss of UNC-31 function blocks the release of neuropeptides and biogenic amines from neurons [46], and loss of UNC-13 function blocks release of the canonical neurotransmitters [48]. We generated *gpa-3;unc-31(e928)* and *gpa-3;unc-13(n2813)* mutants and measured the body fat of the respective single and double mutants. Interestingly, we found that loss of *unc-13* resulted in complete suppression of the fat loss seen in the *gpa-3* mutants (Fig 5A and S5A Fig). This result suggested that rather than neuropeptides and biogenic amines, the canonical neurotransmitters acetylcholine, GABA or glutamate are required for the effects of GPA-3 signaling.

To determine which of the three canonical neurotransmitter pathways are required downstream of GPA-3, we first examined mutants of the presynaptic re-uptake transporters for GABA (*snf-11*), glutamate (*glt-4*) and ACh (*cho-1*). Loss of the re-uptake transporters of the conventional neurotransmitters would disrupt their steady-state levels in the synaptic cleft, and thus indicate a potential role in regulating body fat stores. *snf-1(ok156)* and *glt-4(bz69)* mutants did not appreciably alter body fat stores, whereas *cho-1(ok1069)* mutants had approximately 40% of the body fat of wild-type animals (Fig 5B and S5B Fig), resembling *gpa-3* null mutants. ACh synthesis and breakdown occur via mechanisms distinct from the other neurotransmitters: after release into the synapse, unbound ACh is cleaved to form acetyl-CoA and choline by the enzyme acetylcholinesterase within the synaptic cleft itself. Choline is then taken up into the pre-synaptic neuron by the CHO-1 re-uptake transporter, and this step is a rate-limiting source of choline for presynaptic ACh synthesis. Thus, *cho-1* mutants are defective in the re-uptake of synaptic choline and are deficient in ACh [49, 50]. *gpa-3;cho-1* mutants display similar fat content as either single mutant alone (Fig 5C and S5C Fig). To determine the extent to which changes in ACh signaling regulate body fat stores, we examined the available mutants in the acetylcholinesterase genes, *ace-1*, *ace-2* and *ace-3*, which have increased synaptic ACh [51–53]. Relative to wild-type animals, *ace-1;ace-2* double mutants had a significant increase in body fat stores, whereas *ace-3* mutants did not show an appreciable difference (Fig 5D and S5D Fig). These results suggested that alterations in synaptic ACh result in changes in body fat stores.

To determine the relationship between *gpa-3* signaling and ACh, and to identify the key acetylcholinesterase responsible for the effects of ACh on body fat, we crossed *gpa-3* mutants with the *ace-1;ace-2* mutants to generate each mutant combination, as well as the *ace-1* and *ace-2* single mutants. We found that the *gpa-3;ace-1* mutants fully suppressed the reduced body fat of *gpa-3* single mutants (Fig 5E and S5E Fig), whereas the *gpa-3;ace-2* double mutants did not, and resembled the *gpa-3* single mutants alone (Fig 5E and S5E Fig). *ace-1* mutants also suppressed the transcriptional induction of *atgl-1* seen in *gpa-3* mutants (Fig 5F). Thus, ACE-1 is required downstream of GPA-3 in the regulation of body fat, suggesting that the GPA-3-mediated fat regulatory signal is transmitted from the ADL neurons via the cholinergic pathway.

**Fig 6:**
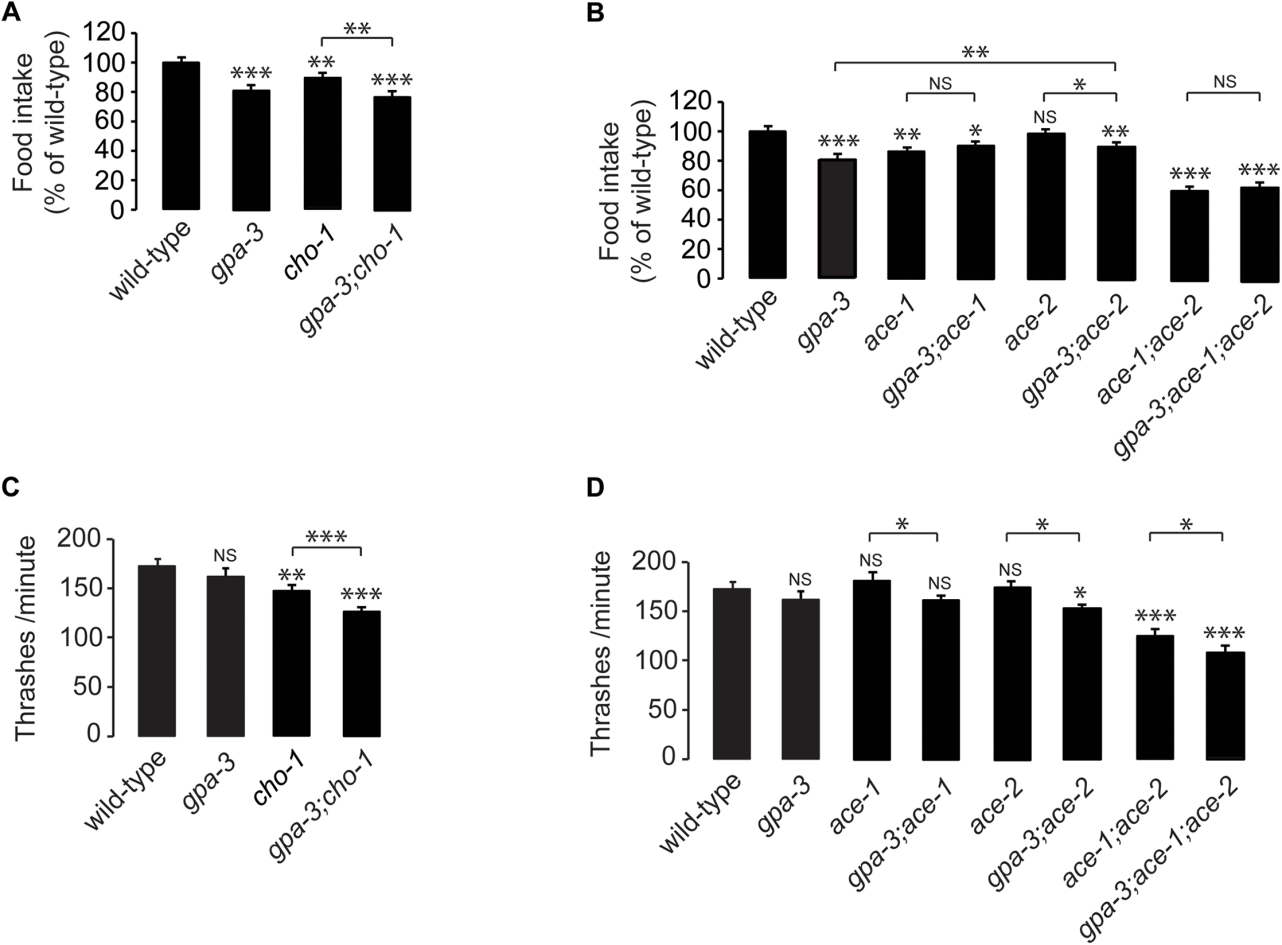
Feeding and locomotion data for mutants of genes required for GPA-3-mediated fat regulation. (A-B) Food intake was measured for each genotype as indicated and is expressed as a percentage of wild-type animals ± SEM (n = 10). NS, not significant; *, p<0.05; **, p<0.01; ***, p<0.001 by one-way ANOVA. (C-D) Thrashing rate was measured for each genotype as indicated and is expressed as number of thrashes per minute ± SEM (n = 15). NS, not significant; *, p<0.05; **, p<0.01; ***, p<0.001 by one-way ANOVA.

We measured food intake and locomotion of the mutants in the cholinergic pathway, with and without *gpa-3* (Fig 6). As described in S3B Fig, *gpa-3* mutants had an ~15–20% decrease in food intake (Fig 6A and 6B). Cholinergic signaling has been known to alter rhythmic behaviors [54, 55], and as expected, *cho-1* mutants also have a significant reduction in food intake, albeit to a lesser extent than the *gpa-3* mutants themselves. *gpa-3;cho-1* double mutants resemble the *gpa-3* single mutants with respect to feeding deficits (Fig 6A). We next examined the *ace* genes with and without *gpa-3* with respect to food intake. *ace-1* mutants have decreased food intake similar to the *gpa-3* mutants, and *gpa-3;ace-1* double mutants do not suppress the *gpa-3* phenotype. Rather, they resemble either single mutant alone (Fig 6B). This is in contrast to the suppression of GPA-3-mediated fat loss, as judged by fat levels as well as by measuring the induction of *atgl-1* by GPA-3 (Fig 5E and 5F). *ace-2* mutants have a negligible effect on food intake, and *gpa-3;ace-2* mutants resemble *gpa-3* mutants alone. Taken together, the *ace-1*-mediated suppression of fat loss of the *gpa-3* mutants is specific, and is not accompanied by suppression of food intake (Fig 6B). Similar results were observed with locomotion (Fig 6C and 6D); additionally, *gpa-3* mutants and *ace-1* mutants do not show appreciable differences in locomotion. Thus, the fat phenotype of *gpa-3* mutants occurs as a selective consequence of a shift towards fat mobilization.

### Pheromone signaling regulates body fat stores via GPA-3 signaling

**Fig 7:**
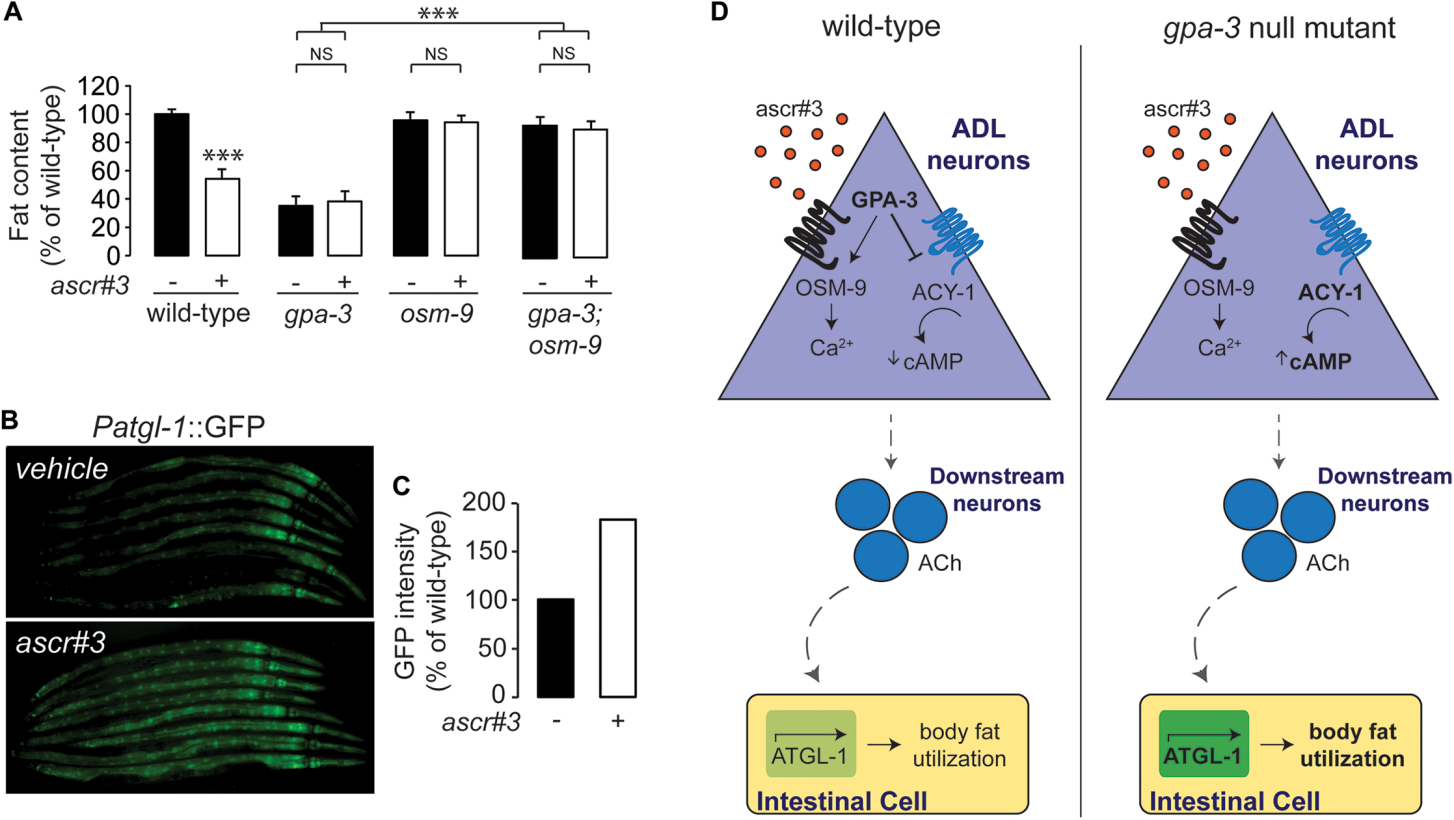
Pheromone signaling regulates body fat stores via GPA-3 signaling. (A) Animals were transferred at L4 to plates containing either ddH_2_O vehicle or 80nM ascaroside, ascr#3, then fixed and stained with Oil Red O. Fat content was quantified for each condition and is expressed as a percentage of vehicle-treated wild-type animals ± SEM (n = 20). NS, not significant; ***, p<0.001 by two-way ANOVA. (B) Representative images are shown of wild-type animals bearing an integrated *atgl-1*::*GFP* transgene exposed either to ascr#3 or vehicle. (C) The fluorescence intensity of *atgl-1* expression was quantified for 8 randomly selected worms for each condition and is expressed as a percentage of wild-type animals exposed to vehicle. (D) Model depicting the ascr#3-mediated regulation of cAMP signaling, via the G protein, GPA-3, in the ADL neurons which controls acetylcholine release in to-be-defined cholinergic neurons, and in turn regulates a stimulatory signal to control body fat stores via the rate-limiting ATGL-1 lipase in the intestine. Under normal conditions, in wild-type animals, environmental levels of the ascaroside ascr#3 regulate the extent to which GPA-3 in the ADL neurons inhibits the downstream adenylyl cyclase ACY-1 and, therefore, controlling levels of cAMP in the ADL neurons. This, in turn, controls the level of acetylcholine released from small, clear vesicles in downstream neurons, and initiates a signal to the intestinal cells to regulate the activity of ATGL-1 (left panel). In *gpa-3* mutants, irrespective of ascr#3 levels, the GPA-3 mediated inhibition of ACY-1 is lost, causing more cAMP to be produced in the ADL neurons. This, in turn, causes more acetylcholine to be released in downstream neurons, ultimately up-regulating ATGL-1 in the intestinal cells, and a constitutive loss of body fat due to increased fat utilization (right panel).

The ADL neurons mediate avoidance behavior from aversive stimuli [56–58] and are also shown to modulate social feeding behavior in response to high O_2_ levels [59]. These effects are mediated predominantly through the TRPV channel, OSM-9 [59, 60]. We found that *osm-9* mutants had wild-type body fat levels, and fully suppressed the reduced body fat of *gpa-3* mutants (Fig 7A and S6 Fig). These results suggested that an aversive function encoded by ADL neurons was related to the GPA-3-mediated fat phenotype. ADL neurons detect an ascaroside pheromone called ascr#3 (also called C9) and initiate an aversive response in N2 wild-type animals that is abrogated in *osm-9* mutants [61]. Pheromone signaling in *C*. *elegans* was originally shown to control developmental fate decisions [62–64]. However, a recent body of evidence has shown that a chemically-diverse family of ascaroside-based pheromones function individually and in combination, to elicit behaviors that collectively transmit population structure and population density information [20, 27, 65]. We wondered whether ascr#3, the ascaroside detected by the ADL neurons would alter body fat stores. Administration of ascr#3 at a dose known to elicit Ca^2+^ transients in ADL neurons [66] led to a robust decrease in body fat stores (Fig 7A and S6 Fig). *gpa-3* and *osm-9* single mutants, and *gpa-3;osm-9* double mutants, did not display a further reduction in body fat upon ascr#3 administration, suggesting that activation of ADL neurons by ascr#3 decreases body fat stores via GPA-3-dependent signaling (Fig 7A and S6 Fig). As expected, administration of ascr#3 also robustly induced *atgl-1* expression in the intestine (Fig 7B and 7C).

We propose a model in which pheromone-mediated regulation of cAMP signaling in the ADL neurons controls acetylcholine release in to-be-defined cholinergic neurons, which in turn regulates a fat-stimulatory signal to control body fat stores via the rate-limiting ATGL-1 lipase in the intestine (Fig 7D). Under normal conditions, in wild-type animals, population-density-dependent levels of the ascaroside ascr#3 regulates the extent to which GPA-3 in the ADL neurons inhibits the downstream adenylyl cyclase ACY-1 thus controlling cAMP levels in the ADL neurons. This, in turn, controls the level of acetylcholine released from small, clear vesicles in cholinergic neurons, and initiates a signal to the intestinal cells to regulate the activity of ATGL-1 (Fig 7D, left panel). In *gpa-3* mutants, irrespective of ascr#3 levels, the GPA-3 mediated inhibition of ACY-1 is lost, causing more cAMP to be produced in the ADL neurons. This, in turn, causes more acetylcholine to be released in downstream neurons, ultimately up-regulating ATGL-1 in the intestinal cells, and a constitutive loss of body fat due to increased fat utilization (Fig 7D, right panel). Receptors for GPA-3 have been identified in the context of the dauer developmental decision [67]. Two G protein coupled receptors, *srbc-64* and *srbc-66* require GPA-3 signaling from the ASK neurons to mediate the dauer decision in response to the dauer pheromone and the ascaroside C6. *srbc-64* and *-66* are not reported to be expressed in the ADL neurons, nor known to be responsive to ascr#3, and therefore likely function via distinct mechanisms.

Our previous work has suggested that food and oxygen are salient environmental cues that regulate body fat stores via modulation of neuronal circuit function [3, 29]. Based on the studies presented here, we now propose pheromone sensing as a third sensory modality that regulates body fat stores. As an animal encounters a new patch of food, it must adjust its metabolism to reflect its environment. A patch of food that contains other worms must necessarily be shared, whereas a patch of food without worms reflects a relatively greater amount of food. We speculate that ascr#3/GPA-3 signaling from the ADL neurons provides *C*. *elegans* a mechanism to discriminate between these distinct environments, and accordingly modulate its metabolism. Our experiments provide the first insights into the molecular mechanisms by which pheromone sensing from the nervous system regulates peripheral lipid metabolism. In future studies, it will be interesting to determine the extent to which these discrete sensory inputs intersect to coordinate body fat metabolism.

## Materials and methods

### Animal maintenance and strains

*C*. *elegans* was cultured as described [68]. N2 Bristol, obtained from the Caenorhabditis Genetic Center (CGC) was used as the wild-type reference strain. The mutant and transgenic strains used are listed in S1 Table. Animals were synchronized for experiments by hypochlorite treatment, after which hatched L1 larvae were seeded on plates with the appropriate bacteria. All experiments were performed on day 1 adults.

### Cloning and transgenic strain construction

Promoters and genes were cloned using standard PCR techniques from N2 Bristol worm lysates or cDNA and cloned using Gateway Technology (Life Technologies). Promoter lengths were determined based on functional rescue and are available upon request. All rescue plasmids were generated using polycistronic GFP. Transgenic rescue strains were constructed by microinjection into the *C*. *elegans* germline followed by visual selection of transgenic animals under fluorescence. For the microinjections, 5–10 ng/μl of the desired plasmid was injected with 25 ng/μl of an *unc-122*::*GFP* or *myo-2*::*mCherry* coinjection marker and 65–70 ng/μl of an empty vector to maintain a total injection mix concentration of 100 ng/μl. In each case, 10–20 stable transgenic lines were generated. Two lines were selected for experimentation based on consistency of expression and transmission rate.

### Triglyceride extraction and quantitation

Triglycerides were extracted from wild-type and mutant *C*. *elegans* as described [3]. Extracted lipids were quantified by liquid chromatography/mass spectrometry on an HP 1100 MSD^TM,^ using a neutral lipid Pheromex Luna C5 column, following the methodology from Nomura and colleagues [69]. Data were normalized to protein, quantified by the Pierce BCA Protein Assay kit.

### Oil Red O staining

Oil Red O staining was performed as described [3]. For Oil Red O experiments in which animals were treated with a non-hydrolyzable cAMP analogue, animals were added to plates containing either M9 vehicle or 20, 200, or 500μM 8-Bromoadenosine 3′,5′-cyclic monophosphate (Sigma Aldrich). For Oil Red O experiments in which animals were treated with ascaroside ascr#3, animals were added to plates containing either ddH_2_O vehicle or 80nM ascr#3. Within a single experiment, roughly 3500 animals were fixed and stained, 100 animals were visually inspected on slides, following which 15–20 animals were imaged for each genotype/condition. All experiments were repeated at least 3 times. Wild-type and *gpa-3* mutants were included as controls for each experiment.

### Thrashing assay

Thrashing rate was measured as previously described [70]. For each animal, a movement where the head and/or tail swung to the other side was counted as one thrash. 15–20 animals were assessed for each phenotype.

### RNAi

RNAi experiments were conducted as previously described [3]. Plates were seeded with HT115 bacteria containing vector or the relevant RNAi clone four days prior to seeding larvae.

### Image acquisition and quantitation

Black and white images of Oil Red O stained animals and fluorescent images were captured using a 10X objective on a Zeiss Axio Imager microscope. Lipid droplet staining in the first four pairs of intestinal cells was quantified as described [3]. We have found that quantification of the anterior intestine reliably captures fat content of the entire intestine. For all *atgl-1*::*GFP* images, an equal number of worms were chosen blindly and lined up side by side. Fluorescence intensity for all chosen worms was quantified for each condition. Images were quantified using ImageJ software (NIH).

### Quantitative RT-PCR

Total RNA was extracted using TRIzol reagent (Invitrogen). Genomic DNA was removed using an RNase-free DNase kit (QIAGEN). cDNA was prepared using a iScript Reverse Transcription Supermix for RT-qPCR kit (BioRad) according to the manufacturer’s instructions. Quantitative PCR was performed using the SsoAdvanced Universal SYBR^®^ Green Supermix according to the manufacturer’s instructions. Data were normalized to actin mRNA. Primer sequences are available upon request.

### DiI staining

Animals of mixed developmental stages were incubated in a 1:200 dilution of DiI stain (Life Technologies) for 3 hours on a rotating rack. After staining, the animals were seeded onto a plate containing an OP50 bacterial lawn and allowed to dry for approximately 30 minutes. Fluorescent images of animals in the L2-L3 larval stages were captured using a 100X objective on a Zeiss Axio Imager microscope.

### Food intake

Food intake was measured by counting pharyngeal pumping, as previously described [71]. For each animal, the rhythmic contractions of the pharyngeal bulb were counted over a 10 s period under a Zeiss M2 Bio Discovery microscope. For each genotype, 10 animals were counted per condition and the experiment was repeated at least three times.

### Statistics

Wild-type animals were included as controls for every experiment. Error bars represent SEM. Student’s t-test, one-way ANOVA, and two-way ANOVA were used as indicated in the figure legends.

## Supporting information

S1 Fig *gpa-3* null mutants exhibit a significant decrease in body fat content. (A) Images of wild-type animals and *gpa-3* mutants fixed and stained with Oil Red O. Animals are oriented facing upwards, and the head and intestinal cells are as marked. Oil Red O stained droplets are indicated. For each genotype, images depict the full range of the observed phenotype. (B) The integrated density of the lipid droplets is used to quantify body fat stores, as described in the Materials and Methods. Graph represents the integrated density values of individual wild-type animals and *gpa-3* mutants. ***, p<0.001 by Student’s t-test. (C) Representative images of wild-type animals and *gpa-3* mutants exposed to vector control or *atgl-1* RNAi fixed and stained with Oil Red O. The model depicts the section of anterior intestine being represented for each genotype and condition.
(TIF)

S2 Fig Increased cAMP levels lower body fat content. (A) Animals were transferred at L4 to plates containing either M9 vehicle or 20, 200, or 500μM 8-Bromoadenosine 3′,5′-cyclic monophosphate (8-Br-cAMP), then fixed and stained with Oil Red O. Fat content was quantified for each condition and is expressed as a percentage of vehicle-treated wild-type animals ± SEM (n = 12). ***, p<0.001 by one-way ANOVA. (B) Model depicting epistatic relationship between the Go/I protein GPA-3 and the adenylyl cyclase ACY-1 for the regulation of cAMP. (C) Representative images of wild-type animals, *gpa-3*, *acy-1(nu329)*, and *gpa-3;acy-1* mutants fixed and stained with Oil Red O.
(TIF)

S3 Fig Restoration of body fat in transgenic *gpa-3* mutants was not associated with a restoration of food intake. (A) *gpa-3* mutants bearing *gpa-3* expression using a 5kb or a 7kb endogenous promoter were fixed and stained with Oil Red O, as indicated. Relative to non-transgenic controls (-, light gray bars), transgenic animals (+, dark gray bars) bearing the *gpa-3* transgene restored body fat content to the same extent, whether driven by the 5kb or 7kb promoter. Data are expressed as a percentage of body fat in wild-type animals ± SEM (n = 12–16). ***, p<0.001 by one-way ANOVA. (B) Food intake for *gpa-3* mutants bearing *gpa-3* expression using a 5kb or a 7kb endogenous promoter was measured. Data are expressed as a percentage of wild-type animals ± SEM (n = 10). NS, not significant; ***, p<0.001 by one-way ANOVA. (C) Food intake for wild-type animals, *gpa-3*, *acy-1(nu329)*, and *gpa-3;acy-1* mutants was measured. Data are expressed as a percentage of wild-type animals ± SEM (n = 10). NS, not significant; ***, p<0.001 by one-way ANOVA. (D) Food intake for *gpa-3* mutants bearing *gpa-3* expression using the indicated promoter was measured. Data are expressed as a percentage of wild-type animals ± SEM (n = 10). NS, not significant; ***, p<0.001 by one-way ANOVA.
(TIF)

S4 Fig Representative images for Fig 4. (A-C) Representative images of all genotypes fixed and stained with Oil Red O.
(TIF)

S5 Fig Representative images for Fig 5. (A-E) Representative images of all genotypes fixed and stained with Oil Red O.
(TIF)

S6 Fig Representative images for Fig 7. Representative images of all genotypes and conditions fixed and stained with Oil Red O.
(TIF)

S1 Table *C*. *elegans* strains used in this study. (TIF)
