# Supplementary figures and images for: Pheromone-sensing neurons regulate peripheral lipid metabolism in Caenorhabditis elegans
Source: PLoS Genet. 2017 May 18;13(5):e1006806. doi: 10.1371/journal.pgen.1006806 (PMC5456406; doi:10.1371/journal.pgen.1006806)

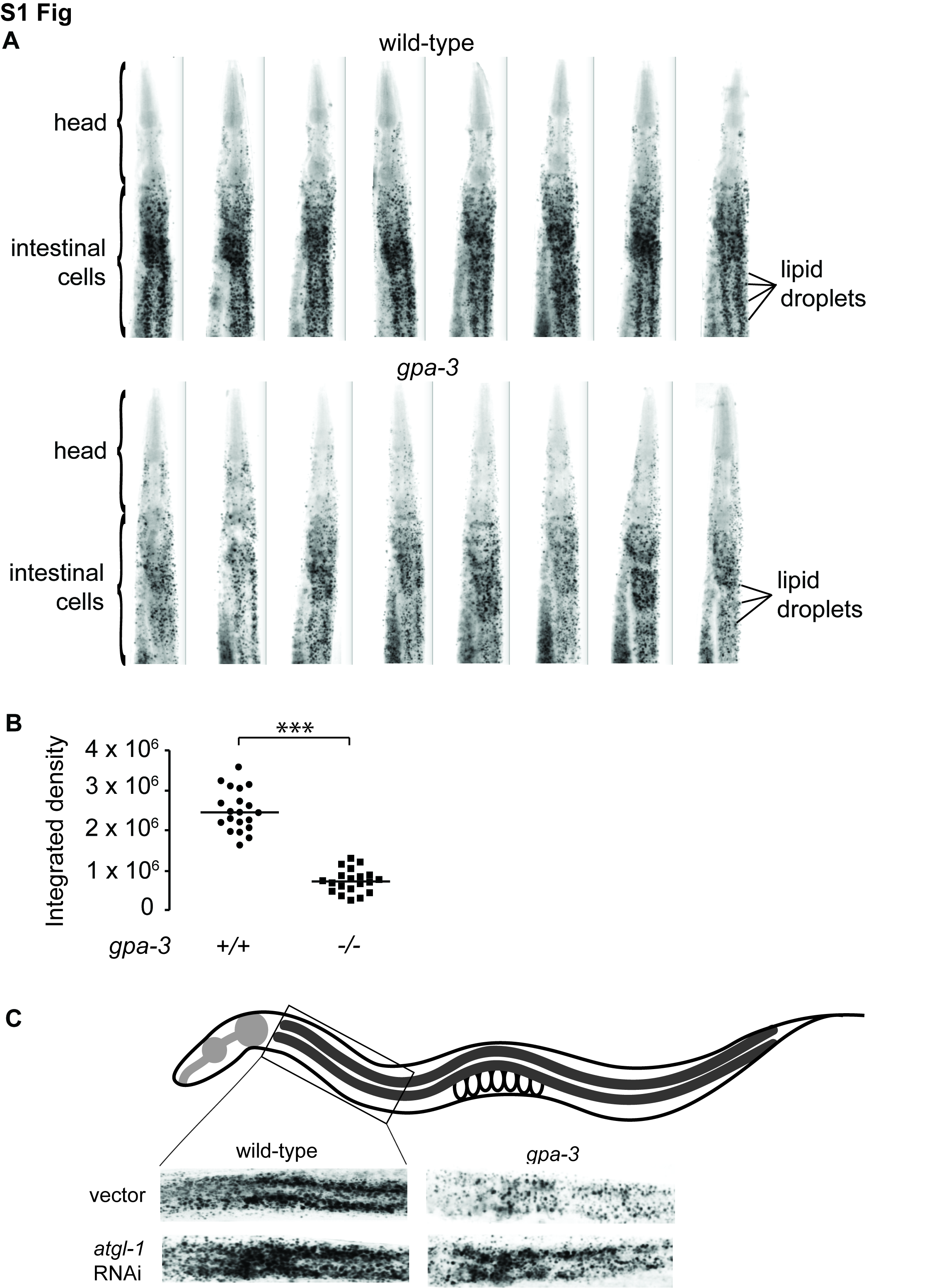

Supplement: S1 Fig — (A) Images of wild-type animals and gpa-3 mutants fixed and stained with Oil Red O. Animals are oriented facing upwards, and the head and intestinal cells are as marked. Oil Red O stained droplets are indicated. For each genotype, images depict the full range of the observed phenotype. (B) The integrated density of the lipid droplets is used to quantify body fat stores, as described in the Materials and Methods. Graph represents the integrated density values of individual wild-type animals and gpa-3 mutants. ***, p<0.001 by Student’s t-test. (C) Representative images of wild-type animals and gpa-3 mutants exposed to vector control or atgl-1 RNAi fixed and stained with Oil Red O. The model depicts the section of anterior intestine being represented for each genotype and condition. (TIF) [file pgen.1006806.s001.tif]

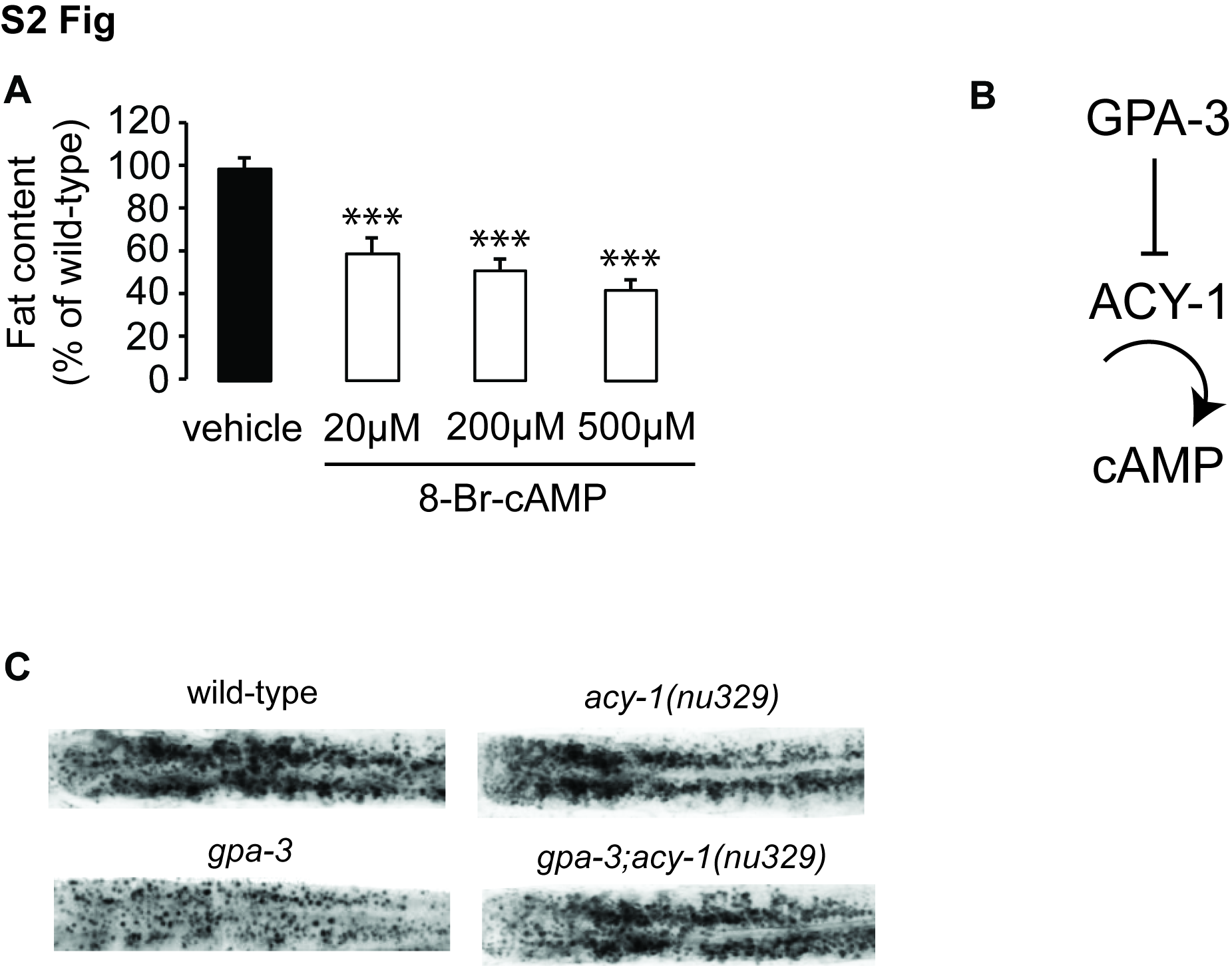

Supplement: S2 Fig — (A) Animals were transferred at L4 to plates containing either M9 vehicle or 20, 200, or 500μM 8-Bromoadenosine 3′,5′-cyclic monophosphate (8-Br-cAMP), then fixed and stained with Oil Red O. Fat content was quantified for each condition and is expressed as a percentage of vehicle-treated wild-type animals ± SEM (n = 12). ***, p<0.001 by one-way ANOVA. (B) Model depicting epistatic relationship between the Go/I protein GPA-3 and the adenylyl cyclase ACY-1 for the regulation of cAMP. (C) Representative images of wild-type animals, gpa-3, acy-1(nu329), and gpa-3;acy-1 mutants fixed and stained with Oil Red O. (TIF) [file pgen.1006806.s002.tif]

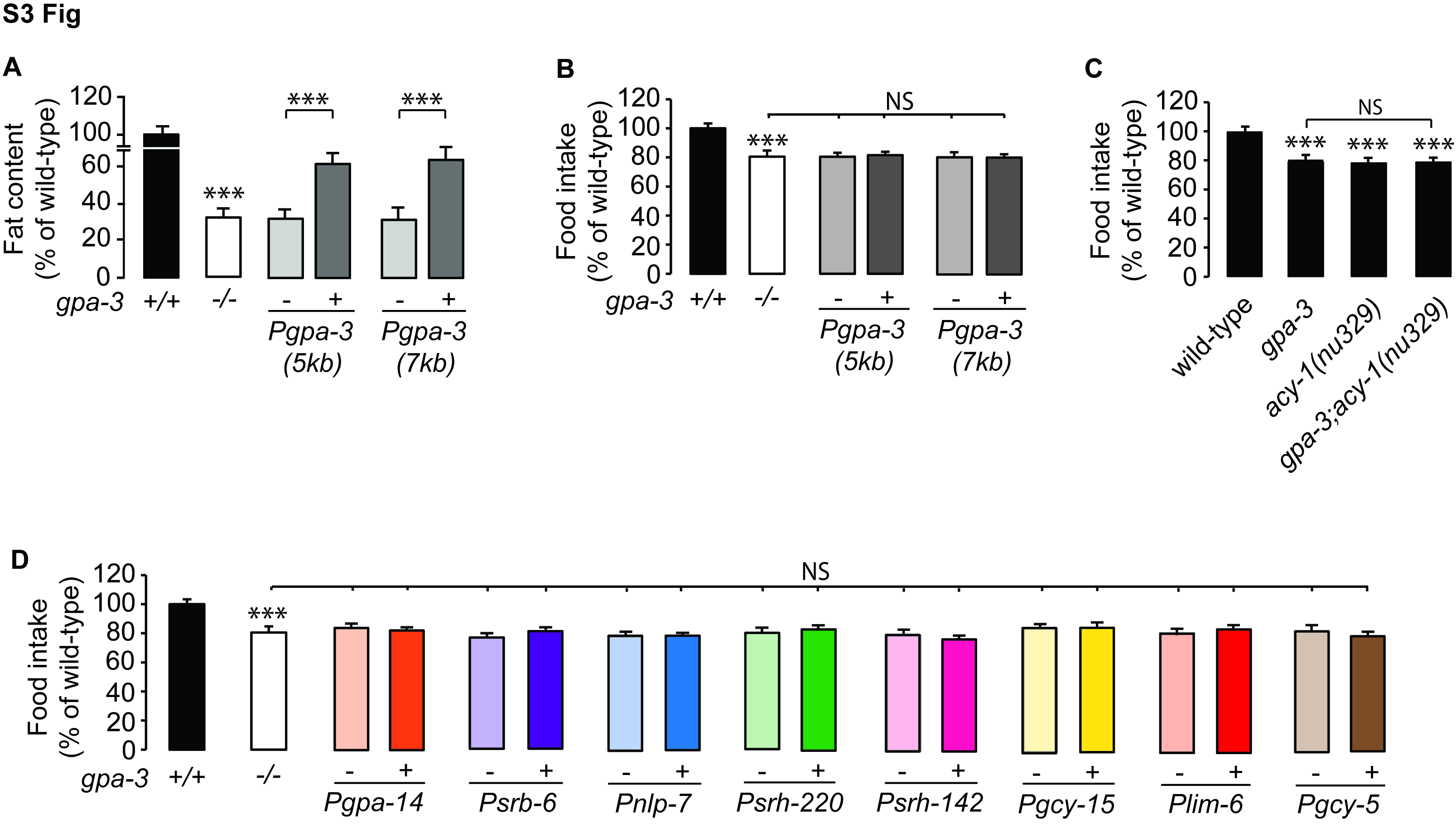

Supplement: S3 Fig — (A) gpa-3 mutants bearing gpa-3 expression using a 5kb or a 7kb endogenous promoter were fixed and stained with Oil Red O, as indicated. Relative to non-transgenic controls (-, light gray bars), transgenic animals (+, dark gray bars) bearing the gpa-3 transgene restored body fat content to the same extent, whether driven by the 5kb or 7kb promoter. Data are expressed as a percentage of body fat in wild-type animals ± SEM (n = 12–16). ***, p<0.001 by one-way ANOVA. (B) Food intake for gpa-3 mutants bearing gpa-3 expression using a 5kb or a 7kb endogenous promoter was measured. Data are expressed as a percentage of wild-type animals ± SEM (n = 10). NS, not significant; ***, p<0.001 by one-way ANOVA. (C) Food intake for wild-type animals, gpa-3, acy-1(nu329), and gpa-3;acy-1 mutants was measured. Data are expressed as a percentage of wild-type animals ± SEM (n = 10). NS, not significant; ***, p<0.001 by one-way ANOVA. (D) Food intake for gpa-3 mutants bearing gpa-3 expression using the indicated promoter was measured. Data are expressed as a percentage of wild-type animals ± SEM (n = 10). NS, not significant; ***, p<0.001 by one-way ANOVA. (TIF) [file pgen.1006806.s003.tif]

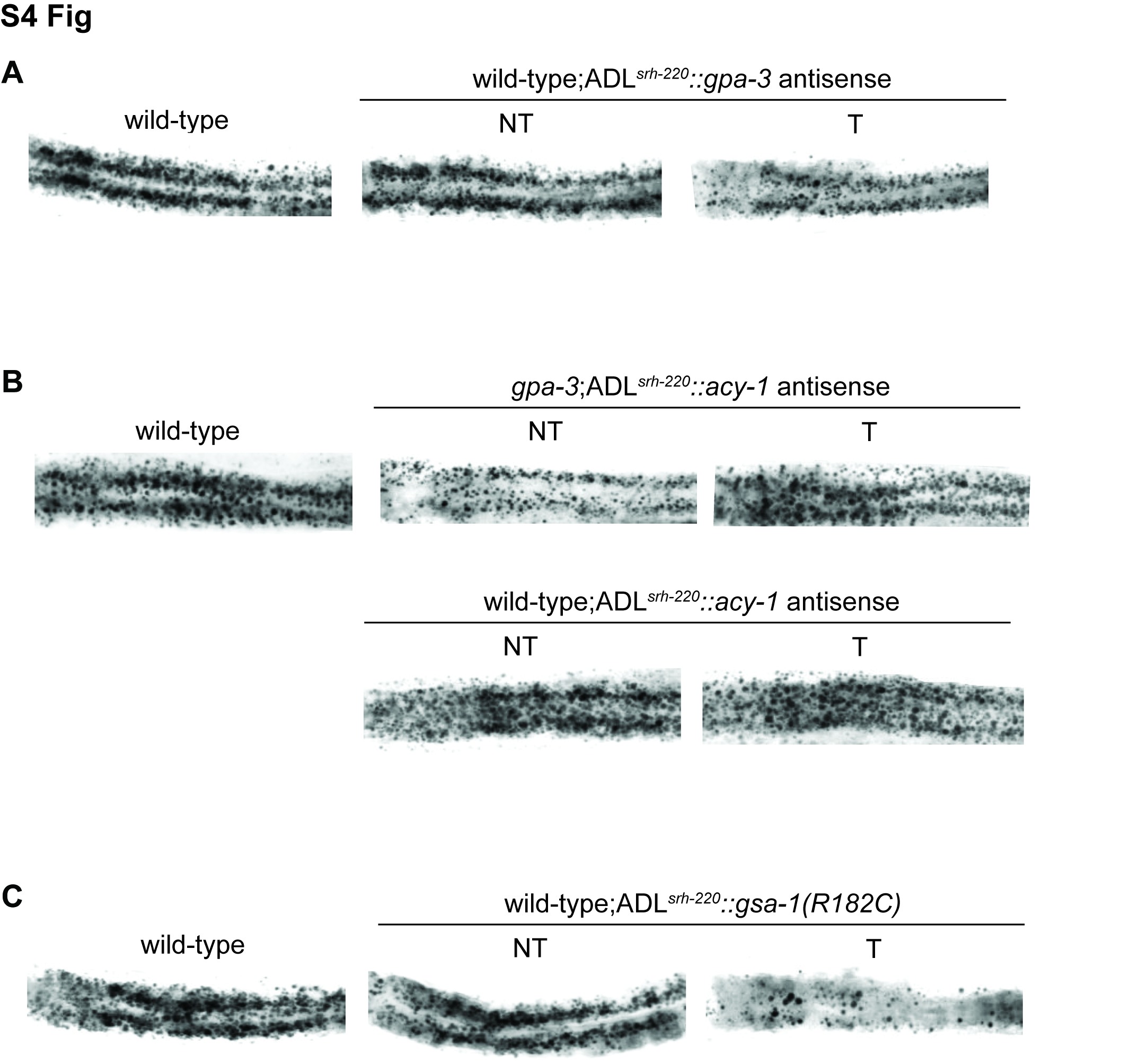

Supplement: S4 Fig — (A-C) Representative images of all genotypes fixed and stained with Oil Red O. (TIF) [file pgen.1006806.s004.tif]

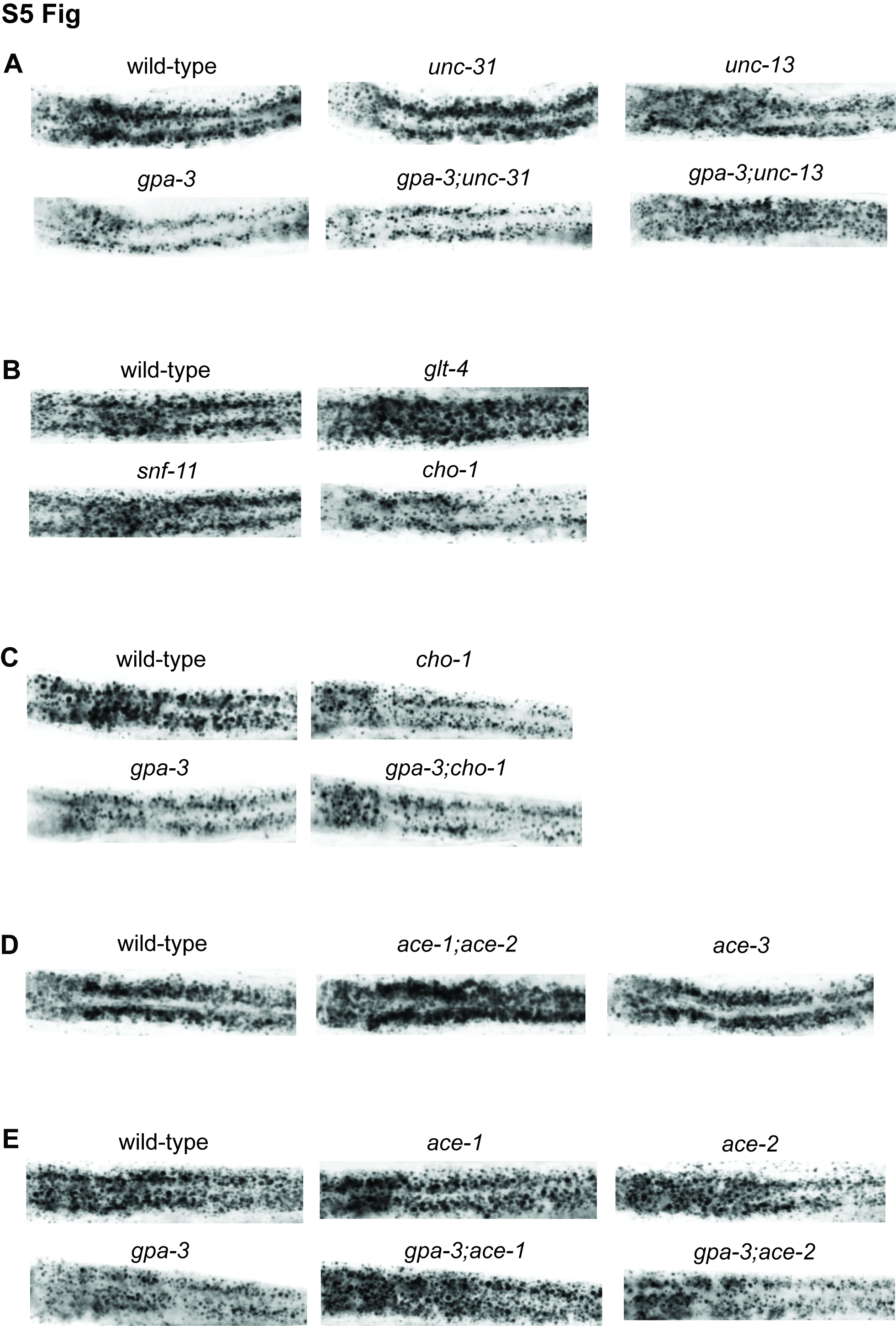

Supplement: S5 Fig — (A-E) Representative images of all genotypes fixed and stained with Oil Red O. (TIF) [file pgen.1006806.s005.tif]

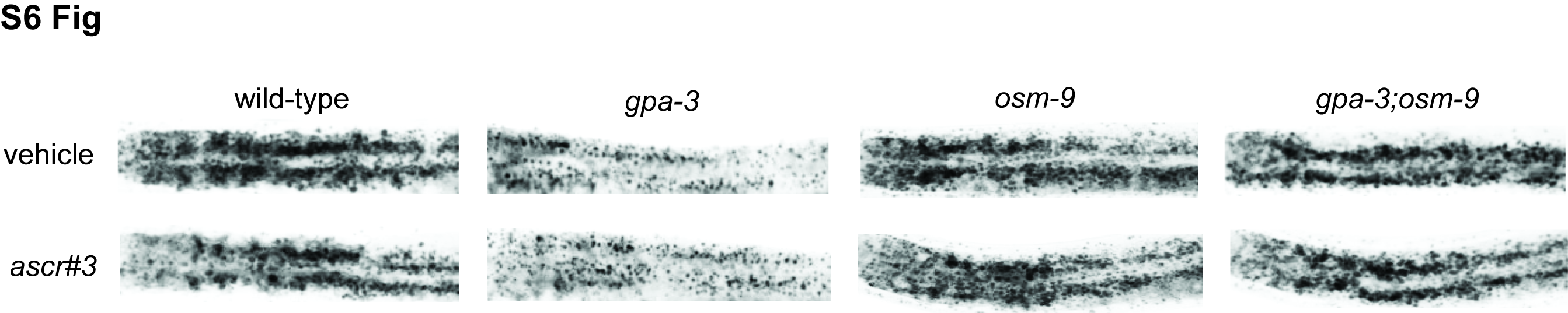

Supplement: S6 Fig — Representative images of all genotypes and conditions fixed and stained with Oil Red O. (TIF) [file pgen.1006806.s006.tif]
